# Obituary

**Published:** 1874-05

**Authors:** 


### OBITUARY.

At a called meeting of the Zanesville Academy of Medicine, held May 2, to take action in relation to the death of its late Fellow, Dr. JNO. G. F. HOLSTON, Sr., which took place at Washington, D. C., May, 1, 1874. The followresolutions were adopted :

That we, whose occuption has been to relieve human suffering, are reminded that the time must come when our places on earth shall be vacted. Therefore,

*Resolved*, That in the death of Dr. Holston, the Zanesville Academy of Medicine loses one of its prominent members, and the profession at large, an eminent physician and surgeon of extensive professional and literary culture, ripe experience and acurate judgment, and society a warm-hearted, genial and generous member, whose life has been mainly devoted to the good of his fellow-beings.

*Resolved*, That we attend the obsequies of our deceased Fellow in a body.

*Resolved*, That we deeply sympathize with the family and relatives of the deceased,

*Resolved*, That the Corresponding Secretary transmit a copy of these resolutions to the family, the city Press and the Medical Journals.

C. C. HILDRETH, Chairman.

A. E. BELL, Secretary pro tem.

Dr. HOLSTON was Prof. of Anatomy in the National Medical College, and attending physician to the presidential household.

